# Transcriptome-Wide Mapping of 5-methylcytidine RNA Modifications in Bacteria, Archaea, and Yeast Reveals m^5^C within Archaeal mRNAs

**DOI:** 10.1371/journal.pgen.1003602

**Published:** 2013-06-27

**Authors:** Sarit Edelheit, Schraga Schwartz, Maxwell R. Mumbach, Omri Wurtzel, Rotem Sorek

**Affiliations:** 1 Department of Molecular Genetics, Weizmann Institute of Science, Rehovot, Israel; 2 Broad Institute of MIT and Harvard, Cambridge, Massachusetts, United States of America; University of Florida, United States of America

## Abstract

The presence of 5-methylcytidine (m^5^C) in tRNA and rRNA molecules of a wide variety of organisms was first observed more than 40 years ago. However, detection of this modification was limited to specific, abundant, RNA species, due to the usage of low-throughput methods. To obtain a high resolution, systematic, and comprehensive transcriptome-wide overview of m^5^C across the three domains of life, we used bisulfite treatment on total RNA from both gram positive (*B. subtilis*) and gram negative (*E. coli*) bacteria, an archaeon (*S. solfataricus*) and a eukaryote (*S. cerevisiae*), followed by massively parallel sequencing. We were able to recover most previously documented m^5^C sites on rRNA in the four organisms, and identified several novel sites in yeast and archaeal rRNAs. Our analyses also allowed quantification of methylated m^5^C positions in 64 tRNAs in yeast and archaea, revealing stoichiometric differences between the methylation patterns of these organisms. Molecules of tRNAs in which m^5^C was absent were also discovered. Intriguingly, we detected m^5^C sites within archaeal mRNAs, and identified a consensus motif of AU**C**GANGU that directs methylation in *S. solfataricus*. Our results, which were validated using m^5^C-specific RNA immunoprecipitation, provide the first evidence for mRNA modifications in archaea, suggesting that this mode of post-transcriptional regulation extends beyond the eukaryotic domain.

## Author Summary

Ribonucleic acids are universally used to express genetic information in the form of gene transcripts. Although we envision RNA as a mere copy of the DNA four-base code, modification of specific RNA bases can expand the information code. Such modifications are abundant in transfer RNA (tRNA) and ribosomal RNA (rRNA), where they contribute to translation fidelity and ribosome assembly. Recent studies in eukaryotes have shown that mRNA modifications such as RNA-editing (conversion of an adenosine base to inosine), N6-adenine methylation (m^6^A), and 5-methylcytidine (m^5^C) can change the coding sequence, alter splicing patterns, or change RNA stability. However, no mRNA modifications in bacteria or archaea have been documented to date. We have used an approach that enables mapping of the m^5^C modifications across all expressed genes in a given organism. Applying this approach on model bacterial, archaeal, and fungal microorganisms enabled us to reveal the modified RNA bases in these organisms, and to provide an accurate and sensitive map of these modifications. In archaea, we documented multiple genes whose mRNAs are subject to RNA modification, suggesting that similar to eukaryotes, these organisms may utilize mRNA modifications as a mechanism for gene regulation.

## Introduction

5-methylcytidine (m^5^C) is a modification that occurs both on DNA and RNA. In eukaryotes, this DNA modification has been extensively studied over the past years, and was found to play a crucial role in genomic imprinting, X-chromosome inactivation, and suppression of repetitive elements [1], [2]. Less is known about the distribution and role of m^5^C sites in RNA. In bacteria, m^5^C positions were described only in rRNA, whereas in archaea and eukaryotes m^5^C was mapped to both tRNA and rRNA. In tRNA molecules, m^5^C sites are typically present at the variable region and the anticodon loop, where they have been shown to stabilize the secondary structure of the tRNA and affect codon identification and tRNA aminoacylation [3]–[5]. For example, m^5^C at position 40 of the yeast tRNA-Phe enables conformational transition of the entire anticodon loop, inducing Mg^2+^ binding at a distant site and resulting in structural stabilization [6]. Absence of an m^5^C modification in the anticodon wobble-base (position 34) of tRNA-Leu in yeast was associated with decreased functionality, observed when nonsense suppressor function was estimated *in vivo* [7]. Recently, it was shown that various modifications on tRNA, including m^5^C, are dynamically modulated during cellular responses to several stress conditions, suggesting that these modifications may play a role in cellular response to stress, potentially by mediating translation rates [8], [9]. In rRNA m^5^C is thought to play a role in translational fidelity [10].

Traditional methods for studying m^5^C and other RNA modifications include HPLC and mass spectrometry, requiring isolation of the specific RNA molecule being studied to near purity [11], [12]. As opposed to the highly abundant tRNAs and rRNAs, isolation of specific mRNA molecules to purity is experimentally challenging, and hence studies of m^5^C modifications on mRNA molecules were limited until recently. Indeed, early studies documenting the presence of m^5^C in mRNA have been controversial; some studies have failed to detect m^5^C in eukaryotic mRNA [13]–[16], whereas others have identified this modification [17]–[19].

Recently, the presence of m^5^C in human HeLa mRNA was studied in a global manner, identifying thousands of potential m^5^C sites within human mRNA [20]. This study utilized RNA bisulfite treatment, which selectively converts C residues, but not m^5^C, into U residues [21], followed by whole transcriptome sequencing. Such bisulfite treatment is commonly used on DNA sequences when DNA methylation states are studied [22], [23]. However, its usage for RNA m^5^C methylation interrogation has been limited until recently. As a result, no study has addressed the presence of m^5^C sites, or any other RNA modification, in prokaryotes in a transcriptome-wide manner, and no modification to bacterial or archaeal mRNA has been described to date.

Here, we set out to generate transcriptome wide maps of m^5^C RNA modification sites in representative model organisms from all three domains of life. We applied bisulfite treatment on total RNA from these organisms, and generated a computational pipeline that identifies methylated positions after accounting for various artifacts. This approach was highly sensitive, and allowed the identification and quantification of the vast majority of known m^5^C positions in tRNAs and rRNAs in these organisms, as well as the detection of novel positions in these structural RNAs. Anti-m^5^C RNA immunoprecipitation confirmed our findings. Intriguingly, we detected m^5^C sites in several mRNAs in archaea, and identified a sequence motif guiding these RNA methylations. This might be suggestive of an additional layer of regulatory complexity present – potentially having functional consequence – on prokaryotic mRNAs.

## Results

**Figure 1:**
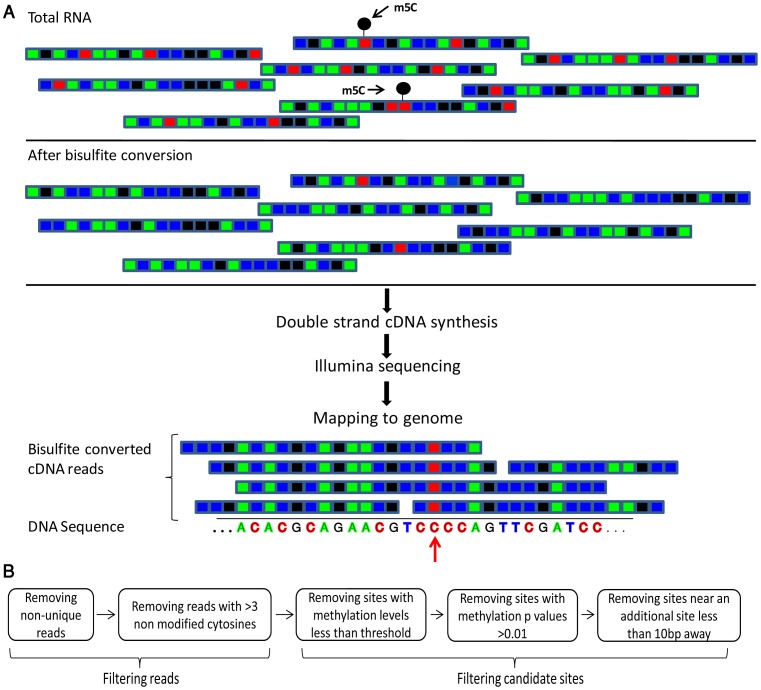
Flow of the bisulfite-treatment/RNA-seq experiment. (A) Shown is a schematic representation of RNA molecules; colored boxes represent single bases. Red, blue, green and black represent C, U/T, A, and G residues, respectively. m^5^C modified cytosine bases are marked by a black circle. Total RNA is bisulfite-treated, leading to deamination of ‘C’ residues into ‘U’, except for methylated residues. Bisulfite-treated RNA is reverse transcribed into cDNA, which is then sequenced via an Illumina HiSeq system. This yields short sequence reads, representing random fragments of the sequenced RNAs. Resulting reads are mapped to the genome using an algorithm that allows mapping of ‘T’ residues in the sequenced cDNAs onto genomic ‘C's. Residues in which ‘C's are found to be consistently non-modified are declared as m^5^C (red arrow). (B) Flowchart of data analysis and artifact filtering (Methods).

To obtain a single-nucleotide resolution overview of m^5^C sites present in RNA, we extracted total RNA from four model organisms, spanning all three domains of life: the eukaryote *Saccharomyces cerevisiae*, the archaeon *Sulfolobus solfataricus*, and the gram positive and negative bacteria, *Bacillus subtilis* and *Escherichia coli*, respectively. We treated the RNA with bisulfite using a modified version of the protocol for bisulfite-treatment of DNA,(described in [21]), prepared a pooled cDNA library from the four organisms, and subjected them to massively parallel sequencing (Table 1; Figure 1A). Since all modified sites identified to date have originated from either tRNAs or rRNAs, we used a protocol that enriches for tRNA and rRNA molecules during this initial approach. This allowed us to test the specificity and sensitivity of the assay (Methods). Sequencing reads from this experiment were obtained primarily from tRNA and rRNA, thus validating the approach. Sequencing results gave sufficient coverage (≥5 reads) for all rRNA molecules annotated for the organisms tested, and for 153 of the 187 (82%) annotated tRNA molecules (Table 1).

**Table 1:** Sequencing reads covering rRNAs and tRNAs in the studied organisms.

Organism	# reads mapped to rRNA	# reads mapped to tRNA	% cytosines converted to uracils^a^	# rRNA	# rRNA genes with mean coverage >5	# tRNA	# tRNA genes with mean coverage >5	% tRNAs covered
*B. subtilis*	4,786,857	42,425	98.3%	2	2	43	32	74%
*E. coli*	206,162	168,557	98.7%	2	2	49	47	96%
*S. solfataricus*	12,654,256	5,626	97.6%	2	2	44	39	89%
*S. cerevisiae*	1,215,559	18,782	98.4%	4	4	51	35	69%

a Conversion rates were calculated out of the raw data prior to filtering.

To identify putative methylated positions, we aligned the reads against genomic DNA that was computationally adjusted so that all C residues were converted to T. Overall, we observed that >98% of all sequenced C residues were converted into T following bisulfite conversion and cDNA sequencing, providing statistical power to identify cytosine residues that reproducibly failed to undergo conversion as putative m^5^C sites (Table 1). For each genomic cytosine, we calculated the number of reads in which that C did not undergo conversion to T, indicative of methylation (Figure 1). For each such position, we determined its “methylation level”, corresponding to the proportion of transcripts in which that position was methylated, and calculated a *p*-value based on Fisher's exact test against the null hypothesis that the position was not methylated (Methods). Based on previous experiments using bisulfite sequencing of DNA [24] and RNA [20], where stretches of non-converted Cs were often found in specific reads (presumably reflecting insufficient exposure of a transcript to bisulfite) we filtered out reads in which 3 or more Cs were not converted into Ts, to avoid assignment of m^5^C modification due to experimental artifacts. Although several modifications other than m^5^C may result in the absence of C->U conversion following bisulfite treatment, we designated all non-converted sites as putative m^5^C sites, for the sake of consistency (see Discussion).

We began by examining methylation sites in tRNAs and rRNAs that passed a statistical metric (*p*<0.01) and in which the methylation levels exceeded 50%. Analysis of the data yielded a total of 10 sites in rRNAs and 85 sites in tRNAs. Below we provide a summary of the identified methylated sites in the different RNA classes in each of the organisms.

### Methylation of rRNA molecules

**Figure 2:**
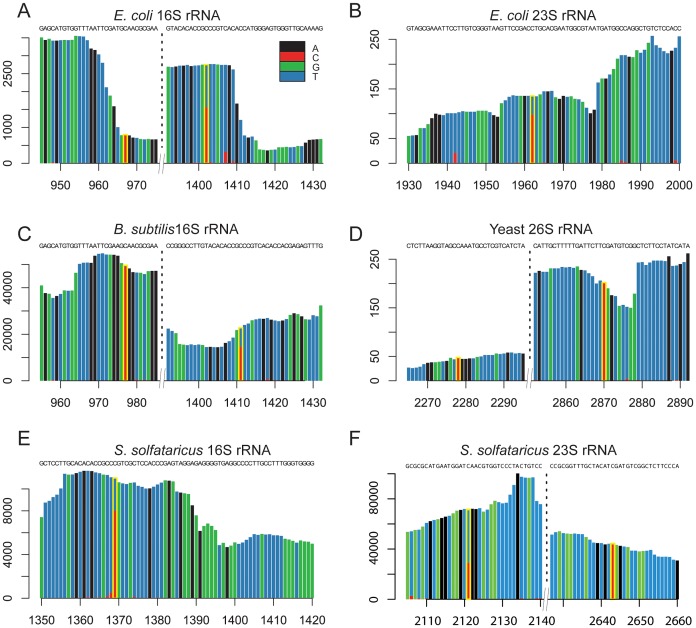
Sites of m^5^C identified in rRNA molecules of the studied organisms. (A–F) Each panel presents a sequence window around a detected methylated site. X-axis, position along the rRNA molecule; Y-axis, number of supporting reads per position. Positions highlighted in yellow are the methylated ones, with the methylation ratio indicated by the fraction of residues in which a “C” residue was not converted into “U”.

In *E. coli* we identified rRNA modifications at 3 distinct positions. The identified residues included position 967 in the 16S rRNA subunit, and position 1962 in the 23S rRNA subunit (Figure 2A–B). Both these residues have been previously determined to contain m^5^C modifications [25]–[27]. In addition, position 1402 in the 16S rRNA of *E. coli* was identified as having a methylation level of 58%. This position is known to harbor a N4,2-O-dimethylcytidine modification which also is not amenable to bisulfite conversion [21], emphasizing the possibility that a fraction of the sites we detected may reflect modifications other than m^5^C (see Discussion).

We identified two modified residues at positions 977 and 1411 of the 16S rRNA of *B. subtilis* (Figure 2C). While neither one of these residues has been previously directly characterized as methylated in *B. subtilis*, they are positionally orthologous to the previously characterized positions 967 and 1402 in the *E. coli* 16S rRNA, respectively (above). Not only were the positions conserved, but also the methylation stoichiometry appeared to be conserved between *E. coli* and *B. subtilis*, with methylation levels of 98.7% at position 977 compared to 97.5% in *E. coli*, and 63.7% at position 1411 compared to 58% in *E. coli*.

**Figure 3:**
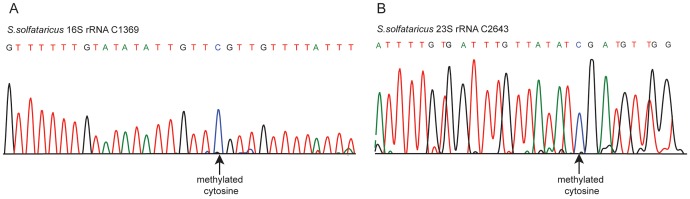
Sanger-based verification of two novel methylated positions in *S. solfataricus* rRNA identified using RNA-seq. Bisulfite-converted sequences were amplified using amplicon-specific primers and sequenced. (A) Position C1369 in the *S. solfataricus* 16S rRNA. (B) Position C2643 in the *S. solfataricus* 23S rRNA.

In *S. solfataricus* we identified one m^5^C site at position 1369 of the 16S rRNA, and another at position 2643 of the 23S rRNA. An additional site with a methylation level of 43% was observed at position 2121 of the 23S rRNA (Figure 2E–F). Position 1369 at the 16S was previously characterized as subjected to an unknown modification [28], whereas no evidence existed for methylation of the 23S sites. Sanger sequencing confirmed that these sites did not undergo bisulfite conversion, providing further support for their methylation (Figure 3). The presence of these modifications is well in line with previous chromatography/mass-spectrometry based analysis, which predicted one m^5^C site in the 16S subunit of *S. solfataricus*, and 1–2 additional sites in the 23S subunit [29].

In yeast we identified two modified positions within the 26S rRNA subunit. Position 2278, which was found to have a methylation level of 72%, was previously reported to contain a methylated site [30]. To our knowledge, we are the first to report the presence of a methylation site at position 2870 (methylation level 98%, Figure 2D). Its presence and the nucleotide composition of this detected site (m^5^C site preceded by a pyrimidine), is consistent with the prediction of an additional m^5^C site in the 26S unit of yeast rRNA, which was not mapped at the time [30].

### Methylation sites within tRNA molecules

**Figure 4:**
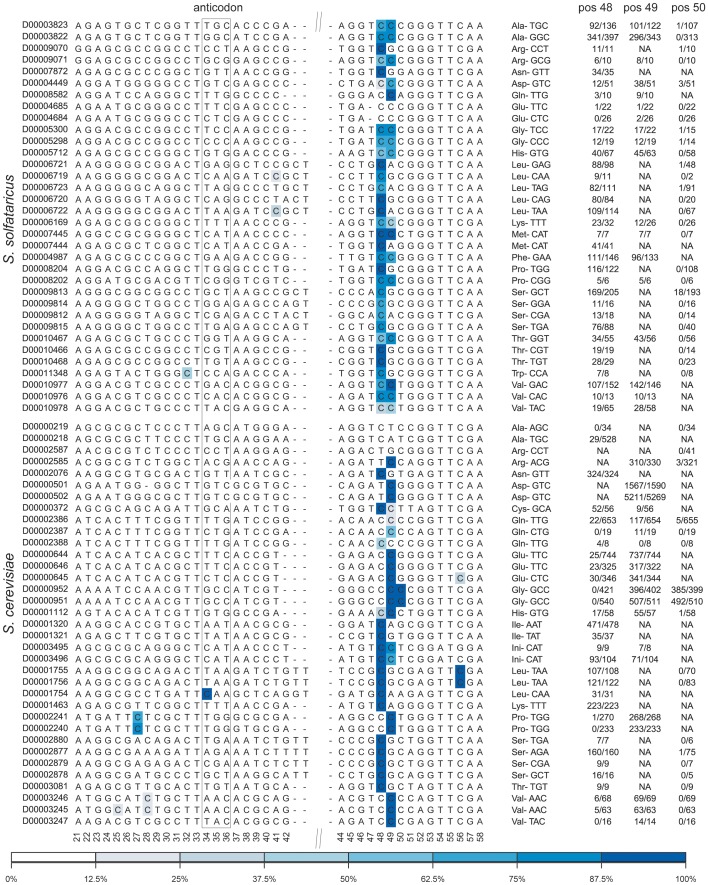
Sites of m^5^C identified in tRNA molecules of the studied organisms. Each line denotes a tRNA sequence, identified (left) by its accession number in tRNAdb [41]. Positions along the consensus are indicated below the sequences, numbers based on *E. coli* tRNA positions as in [31]. The relevant anticodon is given for each tRNA. Methylated positions are color coded by their methylation ratio, according to the presented color gradient (bottom). For positions 48–50, numbers indicate the proportion of reads supporting methylation out of total reads (e.g. 52/56 indicated 52 non-converted reads out of total of 56 covering reads at the specific position). Top-block, tRNAs of *S. solfataricus*; bottom block, tRNAs of *S. cerevisiae*.

Eukaryotic and archaeal tRNA molecules, but not bacterial tRNAs, are known to undergo m^5^C modifications at specific positions. Consistent with this finding, we identified 46 modified sites in 34 tRNAs in *S. solfataricus*, and 39 sites in 30 tRNAs in *S. cerevisiae*. To allow comparative analysis of methylation levels in different tRNAs, we generated a multiple alignment of the archaeal and yeast tRNAs, and color-coded methylated positions along them based on their methylation levels (Figure 4). Notably, in this analysis we examined significant (*p*<0.01) methylated positions even when methylation levels were below 50%, to allow full comparative inspection of the data.

Consistent with previous findings, the methylated positions in both archaea and yeast were clustered at positions 48–49 along the tRNA molecule (numbers based on *E. coli* tRNA positions, as in [31]). In archaea most (30/34, 88%) of the tRNAs were methylated at position 48, while about half of these (14/30) were also methylated at position 49. The pattern of m^5^C modification in yeast was different: usually, either position 48 or position 49 was methylated, with only two tRNAs showing modifications at both positions. Two other tRNAs also contained modifications at position 50 in the variable region.

The relatively high coverage of tens and sometimes hundreds of reads in each tRNA allowed us to quantitatively assess similarities and differences in methylation levels at specific positions (presumably corresponding to the percent of tRNA transcripts modified at those positions). In general, levels of methylation in yeast were significantly higher than those in archaea, with mean values of 96% per position in *S. cerevisiae* as compared to mean values of 82% observed in *S. solfataricus* (Mann-Whitney test, *p* = 3.8e-6). This may imply that the typical single, strongly methylated site in yeast may compensate for the presence of two more weakly methylated sites in *S. solfataricus*. This interpretation, however, needs to be taken with caution, as other factors may account for the observed differences in methylation levels (see Discussion).

Surprisingly, a small fraction of the tRNAs were found to be devoid of m^5^C modifications in the variable region, despite the presence of a cytosine in one of the positions 48–50. These include tRNA-Ala^AGC,TGC^ and tRNA-Arg^CCT^ in yeast and tRNA-Glu^TCC,CTC^ in *S. solfataricus*. This selective absence of the modification in a subset of tRNAs might provide a potential anchor towards elucidating the role of this modification on tRNAs. Alternatively, other types of RNA modifications, not detectible through bisulfite-conversion, may replace m^5^C in these tRNAs.

Several additional methylated positions were observed in our data, including the previously identified methylation in the anticodon (position 34) of yeast tRNA-Leu [32], as well as methylations at position 27 of tRNA-Pro and position 56 of tRNA-Leu. Additional putative m^5^C sites were observed in *S. solfataricus* tRNA-Trp^CCA^ and tRNA-Leu^TAA^, but given their relatively low methylation levels, these sites require further verification. Finally, we failed to detect the known methylation at position 40 of tRNA-Phe [6] due to lack of sequence coverage for this particular tRNA.

### Methylation in mRNA molecules

We were next interested in searching for methylated sites within mRNA molecules. Since our initial experiment lacked sufficient sequence coverage to identify such sites within the vast majority of mRNAs due to the high coverage of rRNA and tRNAs, we performed a second bisulfite-seq experiment on the bacterial and the archaeal RNA using size-selected total RNA (>200 bp) to deplete tRNAs, combined with rRNA depletion protocols to enrich for mRNAs (Methods). For *S. cerevisiae* we made use of a dataset of short sequences previously obtained using bisulfite-treatment of polyA-selected RNA [33].

We used the computational pipeline outlined above to analyze the libraries obtained from the mRNA enrichment protocols. Coverage along mRNA in this experiment was substantially improved, with sufficient coverage (mean coverage ≥5 reads/nucleotide) in 1,394 protein coding genes in *B. subtilis*, 1,110 genes in *S. solfataricus*, and 4,489 genes in yeast. In *E. coli*, due to very high levels of ribosomal RNA reads (>99%), only 287 protein-coding genes had sufficient coverage (Table 2).

**Table 2:** Sequencing reads covering protein coding genes in the studied organisms.

Organism	# reads mapped to coding genes	# coding genes	% cytosines converted to uracils^a^	# coding genes with mean coverage >5 (exp. 1)	# coding genes with mean coverage >5 (exp. 2)	Mean coverage of coding genes^b^	% coding genes covered
*B. subtilis*	1,536,835	4,179	98.9%	1	1,394	21.1	33.4%
*E. coli*	189,572	4,376	98.9%	41	287	14.3	6.6%
*S. solfataricus*	1,216,554	2,979	98.8%	744	2,175^c^	70.6	73.0%
*S. cerevisiae*	5,161,198	6,235	95.8%	1	4,489	72.3	72.0%

a Conversion rates were calculated out of the raw data prior to filtering.

b Relates to mean coverage of all coding genes with mean coverage >5 in experiment #2.

c Data relates to two sequenced lanes combined.

Despite significant coverage of most yeast genes, we were only able to detect a single event of m^5^C on an mRNA in that organism, occurring on a protein of unknown function. No m^5^C events were found in the expressed bacterial mRNAs that were covered in our experiment, although the lack of coverage for most *E. coli* genes prevents us from drawing a strong conclusion as to the possibility of methylated sites in mRNAs for that organism.

**Figure 5:**
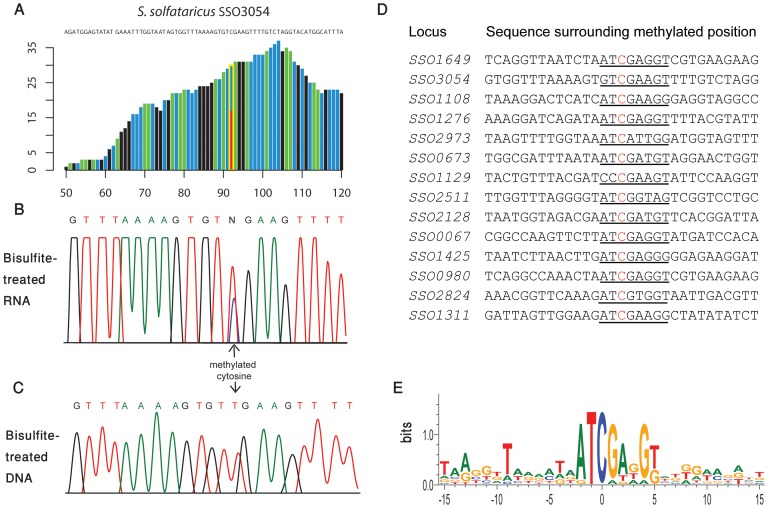
Methylated positions in mRNAs of *S. solfataricus*. (A) Representative position in *S. solfataricus* oxidoreductase gene (locus: *SSO3054*). Colors and axes are as in Fig. 2. (B) Sanger-based verification of the position in panel A, on bisulfite-treated reverse transcribed RNA (C) Sanger-based sequencing of the same position in panel B, on bisulfite-treated DNA, as a negative control (D) Methylated positions in mRNAs of *S. solfataricus* show a consensus motif. Shown are the sequences flanking the methylated positions (red) identified in mRNAs of *S. solfataricus* (Table 3). Recurring sequence motif is underlined. (E) Visual representation of consensus sequence motif, prepared using weblogo [42].

In *S. solfataricus*, the mRNAs of two protein coding genes contained a nucleotide methylated to a level of >50%, and 12 additional genes contained nucleotides with methylation levels of 20–50% (Table 3). Many of the genes harboring the m^5^C modification were enzymes involved in energy and lipid metabolism (notably oxidoreductases and dehydrogenases), which might imply a possible role for this modification in regulating specific metabolic processes. Interestingly, all 14 m^5^C positions in *S. solfataricus* mRNAs were located within a strong sequence motif of AUCGANGU (Figure 5C). This same motif is found at the m^5^C sites we recorded at positions 2121 and 2643 of the *S. solfataricus* 23S rRNA (Figure 2). These results suggest that the same machinery (probably a methyltransferase) is responsible for m^5^C modifications both on the 23S rRNA and on the mRNAs. Moreover, our results imply that this machinery recognizes a consensus sequence of AUCGANGU. The identification of a strong consensus motif reinforces the validity of our bisulfite-sequencing approach in identifying real modified sites on RNA bases, and supports the existence of a single predominant methylation machinery on mRNAs in *S. solfataricus*.

**Table 3:** Modified m^5^C bases in mRNAs.

Organism	Locus tag	m^5^C position	Str	*P* value	# reads covering position	# of reads supporting m^5^C	Meth.%	# reads covering position	# of reads supporting m^5^C	Meth.%	Gene description
					(1^st^ rep)	(1^st^ rep)	(1st rep)	(2^nd^ rep)	(2^nd^ rep)	(2^nd^ rep)	
*S. solfataricus*	*SSO1649*	1501851	+	0.003096	10	7	70.0%	43	23	53.4%	transposase ISC1217
*S. solfataricus*	*SSO3054*	2808514	+	6.19E-07	30	17	56.7%	115	52	45.2%	oxidoreductase
*S. solfataricus*	*SSO1108*	958127	+	8.06E-12	65	31	47.7%	500	179	35.8%	transcriptional regulator Lrs14
*S. solfataricus*	*SSO1276*	1101881	+	4.27E-08	59	22	37.3%	432	103	23.8%	oligo/dipeptide transport, ATP binding protein
*S. solfataricus*	*SSO2973*	2718024	+	4.48E-08	60	22	36.7%	607	169	27.8%	quinol oxidase-2, subunit I/III, cytochrome aa3
*S. solfataricus*	*SSO0673*	578303	−	0.000797	30	10	33.3%	66	21	32.8%	hypothetical protein
*S. solfataricus*	*SSO1129*	971413	+	1.63E-07	70	21	30.0%	417	144	34.5%	heterodisulfite reductase, subunit B
*S. solfataricus*	*SSO2511*	2280456	+	7.51E-09	85	25	29.4%	414	65	15.7%	acyl-CoA dehydrogenase
*S. solfataricus*	*SSO2128*	1955967	+	1.94E-08	88	24	27.3%	637	95	14.9%	pyruvic-ferredoxin oxidoreductase delta chain
*S. solfataricus*	*SSO0067*	55904	−	9.42E-06	59	16	27.1%	178	35	19.7%	30S ribosomal protein S2
*S. solfataricus*	*SSO1425*	1278822	+	0.004575	30	8	26.7%	208	22	10.5%	CRISPR-associated protein, Csx7 family
*S. solfataricus*	*SSO0980*	839015	−	0.000231	50	12	24.0%	146	15	10.7%	transposase ISC1217
*S. solfataricus*	*SSO2824*	2585238	−	0.005056	36	8	22.2%	126	19	15.1%	formate dehydrogenase Alpha subunit
*S. solfataricus*	*SSO1311*	1141274	−	0.001131	46	10	21.7%	251	89	35.4%	enoyl CoA hydratase
*S. cerevisiae*	*YMR178W*	619291	+	2.69E-06	13	12	92.3%	NA	NA	NA	protein of unknown function

To verify that the m^5^C modifications we detected reproducibly appear in *S. solfataricus* mRNAs we sequenced a second bisulfite-treated biological replicate sample of total RNA from *S. solfataricus*. Indeed, all m^5^C sites identified in the first experiment were also detected in the second experiment with similar methylation levels but with higher coverage (Table 3). To rule out the possibility that the consensus motif we identified is intrinsically resistant to bisulfite conversion, we mixed the second *S. solfataricus* RNA sample, prior to bisulfite conversion, with two synthesized 200 nt RNA fragments each harboring a common representation of the consensus motif (either ATCGAGGT or ATCGAAGG; Methods). Despite very deep coverage obtained for the two artificial consensus-bearing RNAs (130,000 and 2.8 million reads, respectively), no evidence for m^5^C was observed within these RNAs, and the percentage of cytosines that were not converted into uracils within the two synthesized consensus motifs was 0.6%, similar to non-methylated residues (Table 2). These results show that the methylation sites we observed in *S. solfataricus* mRNAs do not represent a motif-dependent artifact.

To determine if the presence of this consensus sequence is sufficient to drive methylation, we searched for the motif within all *S. solfataricus* protein coding genes. We identified 75 sites that were covered by at least 5 reads (regardless of *p*-value score). Of these, 31 (41%) were methylated at levels >10%, and 13 (17%) were methylated at levels higher than 20%. These levels are far higher than expected by chance alone: examining all sites (n = 22,665) covered by at least 5 reads, but significantly differing from the consensus sequence, only 5.9% had methylation levels of >10%, and 0.9% of the sites had methylation levels exceeding 20%. These results suggest that the presence of this motif is to a large extent sufficient for recognition and methylation by the putative methyltransferase. However, the varying levels of methylation suggest that additional factors other than the immediate sequence environment – such as RNA availability or more distant motifs – may also play a role in determining methylation levels.

We next examined the relative position of the modified sites within the genes they reside. Methylated bases tended to be localized towards the beginning of the gene (Fig. S1, *P* = 0.07). The apparently non-uniform distribution of modified sites may provide an angle for future elucidation of the function of this RNA modification.

### Direct immunoprecipitation of methylated RNA

Several modifications other than m^5^C may result in an inability to convert C->U following bisulfite treatment, including 3-methylcytidine, N4-methylcytidine, N4,2′-O-dimethylcytidine and N4-acetylated variants [20], [21]. To further examine the nature of the novel modifications we observed in *S. solfataricus* RNAs, we set out to conduct direct immunoprecipitation of modified RNA. For this, we used a monoclonal antibody that specifically binds 5-methylcytosine. This antibody is broadly used to specifically detect m^5^C modifications in DNA (e.g. [34]), but since it was raised against 5-methylcytosine nucleotide conjugated to ovalbumin without the ribose or deoxyribose sugar, it is blind to the DNA/RNA context of the modification and hence binds the RNA form of m^5^C as well. As a control we used a second antibody that specifically binds 5-hydroxymethylcytosine (hm^5^C) but not m^5^C. The hm^5^C modification is similar to m^5^C but carries an additional hydroxyl group on top of the added methyl group. Total RNA of *S. solfataricus* was sheared into ∼100 nt-long fragments (the “input”) and immunoprecipitated using the anti-m^5^C or anti-hm^5^C antibodies. Libraries were prepared from immunoprecipitated as well as non-immunoprecipitated input control RNA fragments, and subjected to massively parallel sequencing on the Illumina platform.

**Figure 6:**
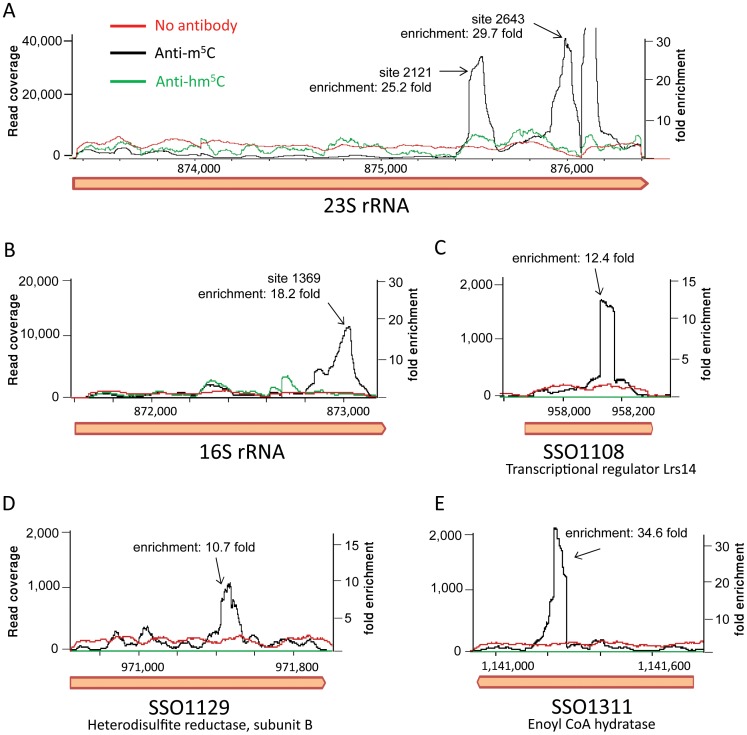
RNA immunoprecipitation with modification-specific antibodies. Shown is the coverage of Illumina-sequenced cDNA following RNA fragmentation, antibody pulldown, reverse transcription and sequencing. Black line, pulldown performed with an anti-5-methylcitosine (hm^5^C) antibody; green line, pulldown performed with an anti-5-hydroxy-methylcitosine antibody; red line, input RNA (no antibody applied). X-axis, position along the genome; Y-axis (right), read coverage of the sequenced anti-m^5^C library; Y-axis (left), fold enrichment of peaks related to median coverage along the gene. The coverage of the anti-hm^5^C and input libraries was normalized using the median of the anti-m^5^C library as a reference point. (A) The 23S gene of *S. solfataricus*. Peaks corresponding to positions 2121 and 2643 in the gene (875,473 and 875,995 relative to the *S. solfataricus* genome, respectively) are marked. Another peak, which did not come up in our bisulfite-based analysis, is observed around position 2760. (B) The 16S gene of *S. solfataricus*. A single peak corresponding to position 1369 in the gene (position 873,040 relative to the genome) is marked. (C–E) Antibody pulldown of m^5^C modifications in protein-coding genes from Table 3.

Mapping the resulting reads to the *S. solfataricus* genome resulted in a highly non-uniform coverage (Fig. 6). Peaks in the coverage were clearly observed in the rRNA at the exact locations identified by the bisulfite sequencing as m^5^C modified (positions 1369 in the 16S and 2121 and 2643 in the 23S). These peaks represented enrichment of 18–30 fold over the rest of the rRNA molecule, but no enrichment was observed in these positions in the non-immunoprecipitated (input) sample or the sample immunoprecipitated by the hm^5^C antibody. Most (10/14, 71%) of the sites we identified in the mRNAs of *S. solfataricus* (Table 3) were also found to be enriched in the immunoprecipitated library (Fig. 6C–E, enrichment of 10–35 fold). These results provide strong independent verification that the sites we identified in the rRNAs and mRNAs of *S. solfataricus* indeed correspond to m^5^C modifications.

## Discussion

We have employed bisulfite treatment of RNA, combined with high-throughput sequencing, to generate a sensitive map of methyl-modified cytosines in four model organisms across the microbial tree of life. Our map not only recovers the vast majority of known sites within tRNAs and rRNAs in these organisms, but also provides a measurement of methylation efficiency for each position, and reveals novel sites within rRNAs and mRNAs.

Several modifications other than m^5^C may result in a lack of C→U conversion following bisulfite treatment [20], [21]. Indeed, and in agreement with previously published data [21], position 1402 in *E. coli* identified in this study is known to harbor an N4,2′-*O*-dimethylcytidine modification, which is known to be resistant to bisulfite conversion. Therefore, the novel modified positions we report in this study might correspond to modifications other than m^5^C. To verify that the novel modifications in mRNAs we identified in *S. solfataricus* indeed represent m^5^C, we utilized m^5^C-specific antibodies that are usually used in pulldown experiments of modified DNA molecules that are an epigenetic marker for gene silencing in mammals. Since these antibodies also bind the RNA form of m^5^C, they formed an ideal tool for independent validation of our bisulfite-based findings. Each of the approaches has its strengths and weaknesses: while the bisulfite conversion provides a single-nucleotide resolution positioning of the modified site, it may also report other types of modifications. The antibody-based approach provides specificity to a single type of modification but with lower resolution of about 100 bp [35]. Therefore, the combination of bisulfite-conversion and RNA-immunoprecipitation provides synergistic results.

In eukaryotes, the chemical modification of mRNAs is emerging as an important factor in the regulation of gene expression. These modifications have been shown to affect mRNA translation, splicing, stability and transport [20], [35], [36]. To our knowledge, our report presents the first evidence for mRNA modification in archaea, opening a window into a possible additional layer of gene regulation in archaea. Further studies are needed in order to elucidate the possible function of these modifications. In particular, profiling of the methylation status across different cellular states, or in response to external stimuli, will allow investigation of the extent to which this modification is dynamically regulated. Knockout studies can provide better understanding of the enzymes involved in mediating this modification across different organisms and will be crucial for understanding its function.

Our identification of a defined sequence motif associated with all mRNA methylated positions in *S. solfataricus* strongly suggests that these modifications indeed occur *in vivo* and are not an artifact of the detection method. This motif, along with the recently reported sequence motif associated with m6A modifications on mammalian mRNAs [35], are the only reported cases of clearly defined linear sequence motifs directing multiple RNA methylation events. Indeed, studies on various tRNA and rRNA modifying enzymes had suggested preference for local RNA structures rather than for a specific sequence [25], [37], [38]. In this respect, it is possible that there is a fundamental difference between tRNA and rRNA molecules, in which modifications are often sequentially added according to folding and maturation levels, therefore demanding structural dependency, and mRNAs, in which the linear sequence may be the most important determinant.

The motif we identified in *S. solfataricus*, AUCGANGU, is also found at the modified positions C2121 and C2643 in the 23S rRNA. Therefore, it is likely that the same methyltransferase responsible for modification of these rRNA residues also methylates the positions we detected in mRNAs. It is possible that the modifications we find on mRNAs might reflect a “spillover” of this enzymes' activity onto non-specific mRNA substrates. However, no methylated site on *S. solfataricus* mRNAs resembles the modified sequence of the 16S rRNA in that organism, suggesting a specific activity for the 23S methylase, but not for the 16S methylase, on mRNA positions. Interestingly, recent work by Squires and coworkers similarly found that m^5^C modifications on human mRNAs are mediated by the RNA methyltransferase NSUN2, previously known to act only on human tRNAs [39].

Our approach not only detects methylated positions in RNAs, but also provides a quantitative measure of the fraction of transcripts harboring this methylation (to which we refer as “methylation level”). The close similarities in rRNA methylation levels of orthologous positions between the gram positive bacterium *B. subtilis* and the gram negative *E. coli* reinforces the validity of this quantitative measure. Nevertheless, this measure should be taken with caution, as factors such as miniscule DNA contamination (which is non-methylated), for example, could contribute noise to this measurement. In addition, bulky modifications (which are prevalent in tRNAs) may hinder reverse transcriptase processivity, and hence, completely modified tRNA molecules may be under-represented in the sequenced data. Therefore, the stoichiometric measurements we recorded might be biased. Methodologies that are not based on reverse transcription should be applied to decisively determine methylation stoichiometry.

An additional limitation of our approach is that it will fail to detect positions methylated at low levels, given the conservative cutoff we set of filtering out rarely methylated positions. Indeed, apart from the positions we described with high methylation levels, there were thousands of additional positions with significant *p* values, but with much lower methylation levels. About 95% of these sites had methylation levels lower than 10%, including many putative positions in rRNAs and tRNAs. As most of these positions have not been previously reported, despite the fact that tRNAs and rRNAs have been extensively characterized, it is highly probable that the majority of these sites represent artifacts from various sources. This notwithstanding, this group contains a single site in the *E. coli* 16S rRNA (position 1407), which is, indeed, known to be modified. This suggests that there may be additional real methylated nucleotides within this group. However, our approach is currently unable to differentiate between rarely methylated positions and experimental artifacts.

The emergence of ultra-high throughput sequence interrogation technologies is revolutionizing research on RNA modifications. Application of such technologies, including RNA-seq and RIP-seq, to study such modifications in eukaryotes has recently revealed that mRNAs carry a plethora of conserved modifications, such as A-to-I [40], m6A [35] and m^5^C [20]. Nevertheless, the field of mRNA modifications is still in its infancy, and even in eukaryotes, where several modifications have already been extensively characterized, their functional consequences are still poorly understood. Our report that mRNAs of archaea also carry such modifications raises the intriguing possibility that mRNA modifications in prokaryotes form an additional layer of gene regulation that has not yet been addressed. Whether the m^5^C modifications are functionally relevant, and whether other RNA modifications also exist on prokaryotic mRNAs, remains to be determined.

## Methods

### RNA samples preparation

*Escherichia coli* (MG1655) cells were grown in LB medium to log phase at 37°C. *Bacillus subtilis* str. 168 cells were grown in LB medium to mid log phase at 37°C. *S. solfataricus* P2 (DSMZ 1617) cells were grown in defined modified Brock's mineral medium with final pH 3.5, and *S. cerevisiae* (BY4741) cells were grown in YPD medium to log phase at 30°C. All cells pellets were suspended in RNAlater (Ambion, AM7022) for 30 min at room temperature. Pre-treatments for RNA isolation included Lysozyme digestion for *E. coli* cells, glass beads vortex for *B. subtilis* and Lyticase digestion for *S. cerevisiae*. Total RNA was then extracted using Tri-Reagent (Molecular Research Center Inc.) according to manufacturer's instructions. RNA samples were treated with Turbo DNA-free kit (Ambion) and rRNA was removed from the *E.coli* and *B.subtilis* samples using MICROBExpress mRNA enrichment kit (Ambion).

### Bisulfite treatment

Bisulfite conversion was performed based on the protocol by Schaefer et al. [21]. Briefly, one microgram of each RNA sample was incubated in DNA protect buffer and bisulfite mix, EpiTect Bisulfite Kit (Qiagen), through 6 cycles of denaturation step at 70°C, followed by a deamination reaction step of 1 hour at 60°C. The RNA was purified from the bisulfite reaction mix using Micro Bio-Spin 6 columns (Bio-Rad) and treated with 0.5M Tris–HCl, pH 9 at 37°C for 1 hour. RNA was then washed on YM-10 microcon (Millipore) 5 times with 0.5 ml ultra-pure water. RNA samples were analyzed using the Bioanalyzer (Agilent) to assess degradation status.

### cDNA synthesis and Sanger sequencing

For all 4 organisms, untreated RNA and bisulfite-treated RNA were used as templates for cDNA synthesis using SuperScript II RT (Invitrogen) and random hexamers according to manufacturer's protocol. cDNA was amplified by PCR (ABgene) using regular primers (for untreated RNAs) and primers specific to cytosine-deaminated sequences (for bisulfite treated RNAs). In order to improve sequencing results, generic M13 sequences (containing all 4 bases) were combined with the 5′ end of all the deaminated primer sequences. PCR products were separated on agarose gel and extracted using gel extraction kit (Qiagen). Sanger sequencing of the PCR product was conducted from the M13 primer sequence.

### Library construction and Illumina sequencing

Equal amounts (50 ng) of each of the four organism's bisulfite-treated total RNA were mixed together. The mixed bisulfite-treated RNA sample was used as a template for cDNA library preparation according to the mRNA-seq Illumina protocol, omitting the polyA-based mRNA purification step. In brief, RNA was first fragmented by divalent cations at 94°C for 5 min. Double stranded cDNA was generated using SuperScriptII and random primers. cDNA was then end-repaired, adenylated and end-ligated to adapters. Following gel separation, a ∼200 bp fragment was gel-purified. The cDNA library was further amplified and sequenced using 40 single-read cycles on a Genome Analyser II (Illumina).

### Alignment of reads

40-nt long reads were aligned using Novoalign (Novocraft Technologies Sdn Bhd, http://www.novocraft.com) to bisulfite-converted genomes of *Bacillus subtilis* (NC_000964), *Escherichia coli* (NC_000913), *Sulfolobus solfataricus* (NC_002754) and *Saccharomyces cerevisiae* (NC_001133-NC001148) downloaded from the NCBI website. Reads that did not align to the reference sequence at their original length, were iteratively trimmed by two base-pairs from the end of the read and then realigned, as long as their length exceeded 35 nt. To allow identification of methylated positions in tRNAs and rRNAs occurring in multiple copies in the genome, reads mapping to multiple regions in the genome were randomly assigned to one such region. Parameters used for the indexing step were “–b”, and for alignment were “-t 60 -h 120 -b 4 -l 35 -s 2 -F STDFQ -r Random -u 6”. For the transcriptome-based study of rRNAs and tRNAs, reads were aligned against a database consisting of unique copies of all fully processed tRNAs obtained from the tRNAdb [41], and against a single representative of each of the 5S, 16S and 23S rRNA genes in each of the four organisms. Identical alignment parameters were set as above. The rational for this approach was based on two considerations: First, to prevent dilution of reads to multiple, identical copies of the same tRNAs and rRNAs, and second, to prevent loss of reads due to differences between the transcriptome and the genome, such as in cases of tRNA introns that are excised during maturation of the tRNA molecule.

### Identification of candidate methylated positions

For each cytosine in the genome in both orientations, the number of reads in which the cytosine underwent conversion to uridine (suggesting that it was not methylated), and those in which it was not converted (suggesting methylation) were counted. To eliminate artifacts of various sources, the following filters were applied: (1) identical reads were considered a single read, to eliminate PCR amplification artifacts, (2) reads with ≥3 unconverted cytosines were eliminated, as they might reflect transcripts that for did not obtain sufficient exposure to bisulfite, (3) only positions with a sequencing quality >20 were counted, to eliminate low-quality positions. In the transcriptome based analysis we did not apply the first filter, since for the highly expressed rRNA and tRNA molecules identical reads generally do not reflect PCR artifacts, but merely reflect the high coverage of these genes. Each position was then assigned a methylation level, equivalent to the proportion of reads in which it was not converted, and a *p*-value determining the significance of the number of converted and non-converted reads was assigned using Fisher's exact test against the null hypothesis that all reads were converted. Identified methylation sites that were within 10 nt of an additional site were discarded. Since many genes are present in the genome in multiple copies, following identification of all significantly methylated positions (*p*<0.01), all positions sharing an identical 31-nt sequence surrounding the identified position were collapsed together into a single sequence. Curation of the data confirmed that this efficiently collapsed together sequences from identical genes. The joint methylation level for each collapsed position was recalculated based on the cumulative number of converted and non-converted reads in its ‘parents’.

### Alignment of tRNAs

For the alignment of tRNAs from tRNAdb [41], a multiple sequence alignment of tRNAs was generated using mafft v6.850b, with the parameters “–maxiterate 1000 –localpair”.

### Analysis of the consensus motif in *S. solfataricus*

The motif AUCG-A/U-G/UG-U/G was searched in all protein coding genes in which the potentially methylated ‘C’ in the center of this motif was covered by at least 3 reads, and methylation ratios were recorded. As a negative control the analysis was repeated taking only motifs with at least three mismatches compared to the consensus (specifically, demanding a “C” at position 0, and that position −2 was not an “A”, −1 not a “T”, and +1 not a “G”). For the analysis presented in Fig. S1, we assembled a dataset of 38 sites harboring a consensus and with a methylation *P* value<0.05. Notably, to gain more statistical power for the downstream analysis, here we did not set a threshold on the minimal methylation level. For each site, its relative position within the gene was calculated, from a scale of 0 to 1 (representing the 5′ and 3′ ends of a gene, respectively). We compared the distributions of these methylation sites to 22,665 controls lacking a consensus site, and obtained marginally significant results (t-test, *P* = 0.07).

### RNA spikes

Two ∼200 bp sequences from the *E.coli* genome, containing the consensus sequence found in *Sulfolobus solfataricus*, were amplified via PCR from the genome of *E. coli* MG1655 (primers: p1_fwd TAATACGACTCACTATAGGGTCATGCACGGTGTCGTTATT; p1_rev AAAGGTTTCCATGTCGAACG; p2_fwd TAATACGACTCACTATAGGGGCTGTGGTGATCAGTGTGCT; p2_rev TGGCGTTGATAAAACTGACG). These amplified sequences were used as template for an in-vitro transcription reaction using MaxiScript kit (Ambion, AM1344) to produce RNA transcripts. These RNA transcripts were spiked into *Sulfolobus solfataricus* total RNA prior to the bisulfite conversion treatment. Reads obtained from this library were aligned separately against the *S. solfataricus* genomes, and against a synthetic genome comprising the two *E. coli* templates. Non-converted cytosines were quantified using the pipeline and filters described above.

### RNA immunoprecipitation

*Sulfolobus solfataricus* total RNA was chemically fragmented (Ambion, AM8740) to an average size of ∼100 bp and was subjected to immunoprecipitation with an anti m^5^C monoclonal antibody (Diagenode, MAb-081-010) or anti-hm^5^C polyclonal antibody (Diagenode, pAb-HMC-050) based on the protocol described in ref [35], using two rounds of IP. The precipitated RNA was used for dsDNA Illumina library construction. Illumina MiSeq sequencing of 30 bp paired-end was used to characterize rRNA modifications. Then, Illumina HiSeq run was conducted on the same library with single-end 50 bp reads to gain enough coverage to analyze the mRNA positions.

## Supporting Information

Figure S1 Position of the modified sites respective to the gene in which they reside. Distribution of modified sites carrying the consensus (n = 38) appears in red; negative set of sites lacking the consensus (n = 22,665) is in blue. The relative position of each methylated site within the gene was normalized to a scale between 0 and 1 (denoting the 5′ and 3′ ends of genes, respectively), indicated by the X axis. Y-axis, distribution density.
(PDF)
