# Supplementary figures and images for: Transcriptome-Wide Mapping of 5-methylcytidine RNA Modifications in Bacteria, Archaea, and Yeast Reveals m5C within Archaeal mRNAs
Source: PLoS Genet. 2013 Jun 27;9(6):e1003602. doi: 10.1371/journal.pgen.1003602 (PMC3694839; doi:10.1371/journal.pgen.1003602)

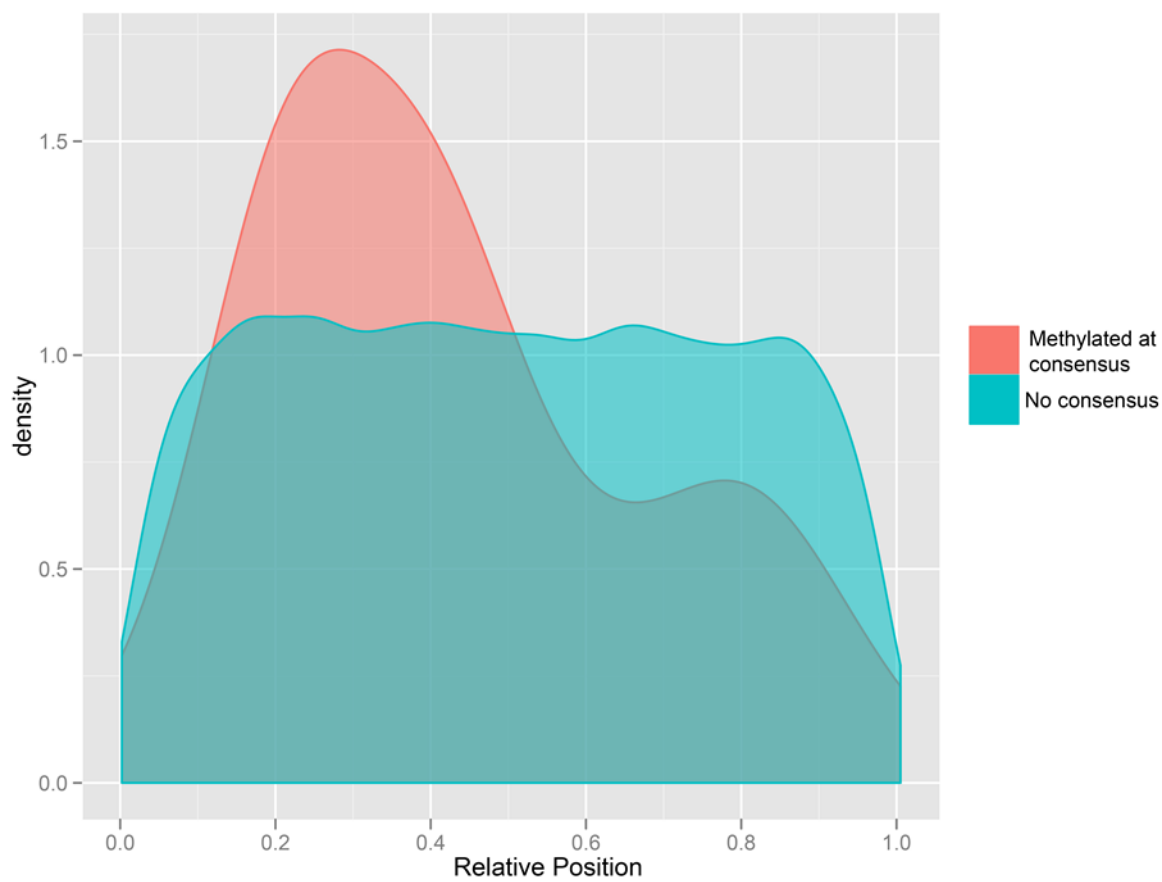

Supplement: Figure S1 — Position of the modified sites respective to the gene in which they reside. Distribution of modified sites carrying the consensus (n = 38) appears in red; negative set of sites lacking the consensus (n = 22,665) is in blue. The relative position of each methylated site within the gene was normalized to a scale between 0 and 1 (denoting the 5′ and 3′ ends of genes, respectively), indicated by the X axis. Y-axis, distribution density. (PDF) [file pgen.1003602.s001.pdf]
